# Post-Effort Changes in Autophagy- and Inflammation-Related Gene Expression in White Blood Cells of Healthy Young Men

**DOI:** 10.3390/cells10061406

**Published:** 2021-06-06

**Authors:** Dorota Kostrzewa-Nowak, Alicja Trzeciak-Ryczek, Paweł Wityk, Danuta Cembrowska-Lech, Robert Nowak

**Affiliations:** 1 Centre for Human Structural and Functional Research, Institute of Physical Culture Sciences, University of Szczecin, 17C Narutowicza St., 70-240 Szczecin, Poland; robert.nowak@usz.edu.pl; 2 Institute of Biology, University of Szczecin, 13 Wąska St., 71-415 Szczecin, Poland; alicja.trzeciak-ryczek@usz.edu.pl (A.T.-R.); danuta.cembrowska-lech@usz.edu.pl (D.C.-L.); 3 The Centre for Molecular Biology and Biotechnology, University of Szczecin, 13 Wąska St., 71-415 Szczecin, Poland; 4 Faculty of Chemistry, Gdańsk University of Technology, 11/12 Narutowicza St., 80-233 Gdańsk, Poland; pawel.wityk@pg.edu.pl

**Keywords:** cell death, chemokines, cytokines, effort, gene expression, inflammation, leukocytes

## Abstract

Acute, strenuous physical exertion requiring high levels of energy production induces the production of reactive oxygen species and metabolic disturbances that can damage the mitochondria. Thus, selective autophagic elimination of defective mitochondria may improve resistance to oxidative stress and potentially to inflammation. The main goal of this study was to evaluate the impacts of intense effort on changes in the expression of select genes related to post-effort inflammation and autophagy. Thirty-five men aged 16–21 years were recruited to the study. The impacts of both aerobic (endurance) and anaerobic (speed) efforts on selected genes encoding chemokines (*CXCL5*, *8–12*) were analyzed. Significant increases in the expression of all studied genes excluding *CXCL12* were observed. Moreover, both types of effort induced an increase in the expression of genes encoding IL-2, -4, -6, -10, IFN-γ and TNF-α, excluding IL-17A. Generally, these efforts caused a significant increase in the relative expression of apoptosis- (*BCL2* and *BAX*) and autophagy- (*BNIP3*, *BECN1*, *MAP1LC3B*, *ATG5*, *ATG7*, *ATG12*, *ATG16L1* and *SQSTM1*) related genes. It seems that the duration of physical activity and its bioenergetic cost has an important impact on the degree of increase in expression of this panel of autophagy-related genes. Anaerobic effort is more strenuous than aerobic effort and requires a higher bioenergetic investment. This may explain the stronger impact of anaerobic effort on the expression of the studied genes. This observation seems to support the protective role of autophagy proposed in prior studies.

## 1. Introduction

It is commonly accepted that physical activity stimulates inflammation [1,2,3,4,5,6], which triggers muscle repair and regeneration [7,8,9]. Physical, cellular and psychological stressors initiate the release of endogenous factors known as danger- or damage-associated molecular patterns (DAMPs) to promote sterile inflammation via the activation of inflammation processes in the absence of exogenous factors such as pathogens [10,11,12,13]. In the context of the sterile inflammation theory [14,15], this mediation can help to better understand the impact of intense stimulating factors on the modulation of the immune system.

Post-intense-stimulus modulation is applied at the physiological and molecular level, including both signaling changes and much more durable epigenetic ones, being one of the main causes of persisting individual variability [16,17]. Intracellular signal transduction results in the activation of transcription factors that regulate gene expression proteins whose autocrine or paracrine secretion develops a cascade of cellular response within a given tissue. The role of cytokine secretion in the cell death pathway is well known [18,19,20].

Numerous cytokines are involved in changes in cell distribution through apoptotic or autophagic pathways [19,20,21]. The Th1-related cytokines, including tumor necrosis factor alpha (TNF-α) and interferon gamma (IFN-γ), promote autophagy, in contrast to Th2-related cytokines (Interleukin 4 (IL-4), IL-13) or anti-inflammatory IL-10 [21]. On the other hand, autophagy enhances the secretion of pro-inflammatory cytokines. This is an important mechanism of regulation of general inflammation induced by various biological factors. The role of Th and Treg cell subsets in modulation of the immune response to endurance effort is well described [22,23,24,25,26]. It is also known that T lymphocyte metabolism is directly controlled by signaling pathways responsible for the activity of these cells and their survival in response to stress stimuli [27].

Macroautophagy, as a protective mechanism, is triggered, among other stimuli, by oxidative stress and hypoxia, which are involved in the removal of oxidatively damaged proteins and organelles [28,29]. This is characterized by the sequestration of large amounts of cytoplasm, long-lived proteins and damaged cellular organelles in double-membraned autophagosomes, which are delivered to lysosomes for degradation, thus regulating homeostasis and promoting cell survival [30]. Acute, strenuous physical effort requiring high levels of energy induces the production of reactive oxygen species and metabolic disturbances that can damage mitochondria, especially during high intensity exercise [31]. Thus, selective autophagic elimination of defective mitochondria may improve resistance to oxidative stress and potentially to inflammation [31,32,33].

The relationship between autophagy and programmed cell death is complex [34]. Autophagy is a cytoprotective mechanism that enables cells to survive adverse conditions and may prevent apoptotic cell death [30,33]. By sequestering damaged mitochondria, autophagy inhibits the formation of apoptosomes by reducing the release of cytochrome c [35]. Disruptions in the process of this selective autophagy lead to an increase in oxidative stress and inflammation, which may induce an apoptotic cascade and elimination of dysfunctional cells [33]. On the other hand, extreme autophagy causes cell death through excessive cell digestion, and may also activate apoptosis [34].

A better understanding of molecular mechanisms involved in post-effort immunomodulation among healthy physically active men might be important to characterizing changes in signaling between cellular components of the immune system that occur as a result of intense endurance (aerobic) or speed (anaerobic) effort stimuli. It is generally accepted that anaerobic effort is more strenuous than aerobic effort and requires a higher bioenergetic investment. Aerobic exercise can be realized as a maximal multistage 20 m shuttle run test (Beep test) [36,37], while anaerobic effort can be achieved during the repeated speed ability (RSA) test [38,39]. Interestingly, our previous study revealed that aerobic (Beep test) and anaerobic (RSA test) efforts, each presenting a markedly different stress stimulus, had a differential impact on the complement system in young physically active men—aerobic effort exerts an immunomodulatory effect among young physically active men whilst anaerobic effort (RSA test) had little influence [40]. Therefore, the main goal of the study was to evaluate the impact of intense effort stimuli on the expression of selected genes related to inflammation and autophagy.

## 2. Materials and Methods

### 2.1. Study Design and Experiment Protocol

Thirty-five healthy men aged 16–21 years, declaring at least 60 min daily physical activity, were asked to perform aerobic and anaerobic physical tasks. For aerobic testing, a maximal multistage 20 m shuttle run test (Beep test) [36,37] was performed. Anaerobic exercise was performed by conducting a repeated speed ability (RSA) test [38,39]. Both tests started with a standardized warm-up consisting of running at a speed of 5 km/h for 10 min and were performed indoors (athletics hall) at a temperature of 20–23 °C, two hours after a light breakfast.

The expression level of genes encoding selected chemokines: *CXCL5*, *CXCL8*, *CXCL9*, *CXCL10*, *CXCL11*, *CXCL12* and interleukins: *IL-2*, *IL-4*, *IL-6*, *IL-10*, *IL-17A*, *IFN-γ*, *TNF-α* as well as autophagy protein genes: *BNIP3*, *BECN1*, *MAP1LC3B*, *ATG5*, *ATG7*, *ATG12*, *ATG16L1*, *SQSTM1* and two apoptosis-related genes *BCL2* and *BAX* in peripheral blood cells triggered by the specified effort during the restitution period were evaluated.

### 2.2. The Participants’ Characteristic

The participants were general morphologically and physiologically characterized before the study according to a protocol described previously [25] and the parameters are presented in Table 1. The body mass, body mass index (BMI), basal metabolic rate (BMR), percentage of fat (FAT), fat mass (FAT MASS), and total body water (TBW) were determined using a Body Composition Analyzer (Tanita BC-418MA, Tokyo, Japan). The cardiorespiratory fitness measures (VO_2_max, VE, AT, RQ, RC, MVV, MET and Rf) were determined using the state-of-the-art breath by breath gas exchange data analyzer Quark CPET (Cosmed, Albano Laziale, Italy) [41].

### 2.3. The Effort Protocol

The Beep test was performed according to standard protocols [36,37]. Briefly, the participants covered 20 m sections, running back and forth over several levels lasting 60 s in increasing (by 0.5 km/h) intensity, as determined by an audible cue with correspondingly shorter intervals. The test started at a speed of 8.5 km/h. It was acceptable to make up any delay in the next 20 m distance. The test stopped after two consecutive failed attempts.

The RSA test was conducted in the morning on a 400 m-long athletics track with an ambient temperature of 20–23 °C [38,39], two hours after a light breakfast. This test consisted of 10 × 15 m sprints starting every 30 s, with a slow walk (active recovery) between repetitions. Participants were instructed to assume the ready position 5 s before starting the next sprint.

### 2.4. The Blood Sampling

Venous blood for the analyses were sampled three times: before the test (pre-test), immediately after the test (not longer than 5 min, post-test) and after lactate recovery (60 min after the test, LA-rec) [42,43]. Peripheral blood samples were collected from the cubital vein. Additionally, to confirm lactate recovery, blood levels of lactate acid were determined. Venous blood samples were collected in a 7.5 mL S-Monovette tube with ethylenediaminetetraacetic acid (EDTA K3, 1.6 mg EDTA/mL blood) (SARSTEDT AG & Co., Nümbrecht, Germany).

### 2.5. The Evaluation of Blood Lactate Level

Lactic acid (LA) concentration was determined with the use of a colorimetric assay kit (PZ Cormay S.A., Łomianki, Poland) according to the manufacturer’s protocol using an Automatic Clinical Chemistry Analyzer (BM-100, BioMaxima S.A., Lublin, Poland). All analyses were verified using a multiparametric control serum and control serums of a normal and a high level (PZ Cormay S.A., Łomianki, Poland).

### 2.6. Isolation of RNA from Blood Samples

Erythrocytes in peripheral blood were lysed with Red Blood Cell Lysis Buffer (RBCL) from A&A Biotechnology (A&A Biotechnology, Gdynia, Poland), using the protocol provided by the manufacturer. After a centrifugation step, total RNA was isolated from the peripheral blood leukocyte pellet, using the GeneMatrix Universal RNA Purification Kit (EURx, Gdańsk, Poland) according to the manufacturer’s protocol. Potential genomic DNA contamination was eliminated by DNase I (EURx) treatment of all RNA samples. The RNA concentration of each sample was estimated using a Qubit Fluorometer (ThermoFisher Scientific, Waltham, MA, USA).

### 2.7. Reverse Transcription

First-strand cDNA of each sample was synthetized from 2 μg of DNase-treated total RNA in a 20 μL reaction volume using the Thermo Scientific RevertAid RT Kit (ThermoFisher Scientific) according to the manufacturer’s protocol. cDNA samples were diluted 10× with nuclease-free water and stored at −20 °C until further analysis.

### 2.8. Real-Time PCR Protocol

Amplification of selected genes from a cDNA template was performed by qPCR using PowerUpTM SYBR^®^ Green Master Mix (Aplied BiosystemsTM, ThermoFisher Scientific) on a CFX96 Touch Real-Time PCR Detection System (Bio-Rad, Hercules, CA, USA). The primers used in the RT-qPCR analyses are listed in Table 2. Cycling conditions (temperature and time) were determined according to the manufacturer’s instructions, taking into consideration melting temperatures of primers and length of expected amplicons. Additionally, to exclude nonspecific products, melting curves of PCR products were analyzed after termination of the amplification reaction. *RACK1* gene was used as a reference gene, according to the studies in Silver et al. [44] and Zhang et al. [45]. A gel representative of the size of the amplification products is presented in Supplementary Material (Figure S1). The relative expression of each analyzed gene normalized to the reference gene (2^−ΔCt^) was calculated for each time point. In addition, Livak’s comparative method [46] was used to calculate the fold change in gene expression normalized to reference gene, relative to pre-test control results (post-test/pre-test ratio and LA-rec/pre-test ratio, respectively). Each sample was analyzed in triplicate to increase the precision of the data obtained.

### 2.9. Statistical Analysis

Statistical analysis was performed using Statistica version 13 software (2017; TIBCO Software Inc., Palo Alto, CA, USA). The normality of the data distribution was assessed using the Shapiro–Wilk test. Normally distributed measure values were presented as mean ± SD, except for age, which was presented as a mean (range). For data with a non-normal distribution, non-parametric statistical tests were used. The significance of differences observed between analyzed time points (pre-exercise versus post-exercise versus lactate recovery) was calculated using Friedman’s analysis of variance for repeated measures followed by a post hoc Dunn’s test with Bonferroni correction. The significance of differences in analyzed parameters between the Beep and RSA tests was calculated using the Mann–Whitney U test. Each time, *p* < 0.05 was considered as a significant difference.

## 3. Results

Baseline characteristics of the participants included in the study are presented in Table 1. The participants’ declared physical activity was 10 years (median; interquartile range 9–12 years). Cardiorespiratory fitness measure results are presented in Table 3. The recovery blood LA results confirmed a return to the pre-test state within 60 min after the effort stimulus for all participants. However, the post-test LA levels differed following the physical efforts induced by the Beep and RSA tests, respectively, implying bioenergetic differences of the tests.

Both aerobic (endurance) and anaerobic (speed) efforts triggered significant increases in the expression of all studied chemokine encoding genes, with the exception of *CXCL12* (Table 4). However, the relative changes in post-test gene expression were significantly different when comparing the types of effort to baseline values for most genes, excluding *CXCL11* and *CXCL12*. The anaerobic (RSA test) exercise caused a significantly higher increase in the post-effort expression of *CXCL*s genes than the aerobic stimulus (Beep test) (Table 4). The lactate recovery expression levels of the *CXCL5*, *CXCL9* and *CXCL10* genes were significantly higher than baseline values in the case of the RSA test group, while in the Beep test group these changes were found only for the expression of genes *CXCL10–12*. The expression ratio of the studied genes calculated between the start time and the LA-recovery time point revealed that the expression of all genes except *CXCL11* was significantly higher in the RSA group than in the Beep group (Table 4).

The effort stimuli that we studied induced an increase in the expression of cytokine encoding genes *IL2*, *IL4*, *IL6*, *IL10*, *IFNG* and *TNF*, but not *IL17A* (Table 5). A significant deviation from starting values in the expression of these genes was observed at the LA-recovery time point. The recovery level of gene expression was significantly lower than the post-effort level in the case of *IL2*, *IL4* and *IL6* in the Beep group, and in the case *of IL2*, *IL6* and *IL10* in the RSA group (Table 5).

The anaerobic effort stimulus (RSA test) was associated with significantly higher post-effort and recovery expression ratios than the aerobic endurance effort (Beep test) (Table 6). In general, both types of effort caused a significant increase in the relative expression of apoptosis- (*BCL2* and *BAX*) and autophagy- (*BNIP3*, *BECN1*, *MAP1LC3B*, *ATG5*, *ATG7*, *ATG12*, *ATG16L1* and *SQSTM1*) related genes (Table 6). Although both types of effort caused significant increases in the expression of studied genes, the anaerobic effort (RSA test) increase was more significant than the aerobic increase (Beep test).

## 4. Discussion

Aerobic and anaerobic efforts induced by running protocols, due to their biomechanical and bioenergetic nature, create a good model for studying sterile inflammation and factors inducing systemic inflammation, as opposed to local inflammation (e.g., viral or bacterial in origin). These two types of effort trigger widespread metabolic change, including in the muscle tissue, nervous system and blood cells, and on the cardiorespiratory and whole system levels. From this point of view, the regulation of gene expression observed in peripheral blood leucocytes is one of the key biological responses to systemic stress factors appearing as a result of strenuous physical effort.

### 4.1. The Impact of Aerobic and Anaerobic Efforts on the Expression of Genes Encoding Selected Chemokines

Immune cells continuously surveil peripheral tissues and respond to migratory signals, attracting them to infiltrate the site of inflammation. Despite the central role of chemokines and their receptors in cell migration, it must by pointed out that it this is a complex multistep process, involving numerous proteins and interactions. Chemokines are signaling molecules responsible for the recruitment of the immune cells to contracting skeletal muscles during physical effort. Post-effort recruitment of NK and T cells is also related to paracrine and autocrine secretion of cytokines following appropriate chemokine secretion [23,24,25]. 

Nedachi et al. [47] evidenced that the production of chemokines CXCL1 and CXCL5 was augmented immediately following electrostimulation of highly developed contractile myotubes. It was evidenced in our study that physical effort, both aerobic and anaerobic, triggered increased expression of genes encoding chemokines *CXCL5*, *8* and *9–11*. CXCL9, 10 and 11 are the agonists of the CXCR3 receptor located on the cellular surface of Th1 cells and antagonists of the CCR3 receptor on Th2 cells [48,49]. This finding confirms the role of Th1 cells in the post-effort immune response observed in young physically active men [24,25].

It has been established that Beclin-1, the product of the autophagy gene *BECN1* increases the infiltration of functional natural killer (NK) cells into the muscle tissue [50,51], NK cells being the most strongly recruited cells after a progressive endurance effort [23]. The literature indicates that, like Beclin-1, other autophagy-related proteins, including Atg5, p62/SQSTM1 and chloroquine, which pharmacologically inhibits autophagy, also induce the expression of selected chemokines, e.g., CCL5 in melanoma cells [51]. On the other hand, CXCL10 has been shown to induce the migration of NK cells [50].

Our results indicate that the increase in expression of autophagy genes (*BNIP3*, *BECN1*, *MAP1LC3B*, *ATG5*, *ATG7*, *ATG12*, *ATG16L1* and *SQSTM1*) in peripheral blood cells was a biological effect of intense physical effort. This observation is in line with the increase in the expression of cell migration-related molecules, including CXCL5, 9, 10 and 11. CXCL12 recruits immune suppressive cell subsets and is responsible for the recruitment of Treg cells [52]. Significant relative change in expression of the gene encoding this protein was observed in response to both aerobic and anaerobic effort in our study. Interestingly, our previous study evidenced a significant role of Treg subsets in regulating the post-effort inflammation status of young physical active men [23,24,25]. The most probable explanation of this phenomenon is that the observed effect is restricted to protein activity without changes in the expression of the *CXCL12* gene between the studied time points. 

The molecular mechanism of CXCL8/IL-8 is related to the activation of transmembrane receptors of CXCRs and the activation of mitogen activated kinase (MAPK) [53]. CXCL8/IL-8 signaling in endothelial cells is a potent pro-angiogenic stimulus, as well as vascular endothelial growth factor (VEGF) [48]. CXCL8 autocrine activation of CXCR2 has been described as one of the mechanisms inhibiting stress-induced apoptosis [49]. Peske et al. [54] suggest that IL-8 and other chemokines may stimulate T cell recruitment, extravasation and migration into peripheral tissues. These processes are selectin and integrin binding, and chemokine signaling-dependent [54]. Our study confirms that physical effort causes an increase in the expression of the *CXCL8/IL8* genes after both aerobic (endurance) and anaerobic (speed) effort. Those observations suggest that the role of CXCL8/IL-8 is homeostatic and probably inflammation protective. The post-effort increase in IL-8 reveals this interleukin as being an important signaling factor involved in restoring immune balance in young men [23,24,25].

### 4.2. The Impact of Aerobic and Anaerobic Effort on the Expression of the Genes Encoding Selected Cytokines

The post-effort inflammation status of physically active men is explained by the secretion of pro- and anti-inflammatory cytokines [23,24,25,55]. Our previous study indicated that progressive effort is related to Th1-related cytokine secretion. It was also evidenced that post-effort increases in IL-10 and TNF-α represent a biological effect [23,24,25,55], most likely related to an inflammatory response to muscle damage caused by exhaustive endurance effort [56]. IL-10 also constitutes an anti-inflammatory response to exercise [57,58], playing a crucial role in muscle tissue regeneration [59,60,61]. These observations were echoed in this study at the gene expression level. 

The participation of IL-2 in lymphocyte T activation and proliferation [18,19,62] relates to its anti-apoptotic role [62,63]. Autocrine signalization of the CD25 receptor and ATK kinase activity impact Bcl-2 protein expression [62]. It seems that increased expression of *IL2* and both *BCL2* and *BAX* genes is potentially involved in restoring the post-effort immune balance. On the other hand, the pro-apoptotic roles of IL-4 and IFN-γ seem to be in line with the significant increase in the post-effort and recovery expression of genes encoding those cytokines and apoptosis-related proteins like Bcl-2 and Bax. The pro-apoptotic role of IL-4 is dependent on IL-2 level [18,64]. In turn, the IFN-γ mechanism of action is related to the control of procaspase-8 expression mediated by STAT proteins in Th lymphocytes. The relative level of *IL2* and *IL4* gene expression observed in our study indicates a shift of the molecular mechanism towards utilizing pathways involving IL-4 and INF-γ. This observation may explain the significant increase in the expression of pro-apoptotic genes like *BAX*.

Autophagy is one of numerous mechanisms underlying the regulation of the inflammatory response during acute exercise. Pro-inflammatory cytokines such as TNF-α, IFN-γ and IL-1α/β activate the process of autophagy, which acts in a negative feedback loop, stopping excessive production of the pro-inflammatory factor due to its ability to suppress the activation of inflammation [21]. This may reflect the ability of autophagy to remove damaged mitochondria that release reactive oxygen species (ROS) [31,32,33]. In turn, cytokines such as IL-4 and IL-10 inhibit autophagy. IL-4 counteracts IFN-γ-induced cellular autophagy and activation of the AKT/mTOR cascade [65,66].

At this peripheral level, autophagy in blood lymphocytes during exercise may be inhibited by the anti-inflammatory environment. However, the main inhibitor of autophagy, mTOR kinase, is also a cellular energy sensor regulated by exercise, and serves in the immune system to activate glycolytic pathways in many leukocyte subtypes to enhance inflammatory and effector functions [67,68]. The results of our study seem to evidence that both aerobic (endurance) and anaerobic (speed) efforts shift the immune balance through the cell death mechanism, at least at the gene expression level.

### 4.3. The Impact of Aerobic and Anaerobic Effort on the Expression of Genes Encoding Selected Autophagy-Related Proteins

Immune activation is linked to oxygen demand and biosynthesis, and immune cells must engage in metabolic reprogramming to generate enough energy to meet these needs [69]. However, acute strenuous physical exertion requiring high levels of energy production induces the production of ROS that can damage mitochondria, especially during high intensity exercise [31]. Autophagy is a cellular mechanism induced, among others, in response to oxidative stress and hypoxia. It is involved in the removal of oxidatively damaged proteins and organelles [28,29], and may be involved in a protective “surveillance” mechanism of leukocyte activity during acute, strenuous exercise. HIF-1α, a transcription factor regulating oxygen balance, is recognized as the main regulator of hypoxia signaling in cells [70,71]. In the hypoxic environment, HIF-1 is activated, and the abundant accumulation of the HIF-1α subunit can initiate the transcription of various low oxygen response genes, including autophagy-related genes, and induce the expression of target genes [70,71], thereby initiating wide ranging adaptation.

HIF-1α has been shown to regulate the activity of mTOR kinase, which is the main inhibitor of the autophagy process, and is also responsible for inducing the transcription of the gene encoding the BNIP3 protein—a key factor regulating mitochondrial autophagy [70,72]. Our study showed increased post-effort expression of the BNIP3-encoding gene after both types of physical effort, but the anaerobic effort caused a significantly higher increase. By binding to Bcl-2/Bcl-xL, BNIP3 inhibits the interaction of these proteins with Beclin-1 [70,72], thereby inducing autophagy and mitochondrial degradation in response to hypoxia. Furthermore, BNIP3 has been shown to play an important role in the constitutive expression of Beclin-1 and Atg5 [71].

It should be noted that the regulation of autophagy is a complex process, controlled by the coordinated action of genes regulated by autophagy [21]. Therefore, to investigate how different types of exercise induce the autophagy process in leukocytes, we measured change in the expression of genes encoding proteins involved in distinct molecular events that lead to autophagous vesicle formation, specifically initiation (Beclin-1), maturation (conjugation systems LC3 and the Atg12-Atg5-Atg16L1 complex), as well as the Atg7 protein which regulates the formation of these elongation systems, and the p62/SQSTM1 protein, which is an adapter of selective autophagy [21,29,30].

Beclin-1 is a protein that plays a key role in the initiation of sequestration, participating in the step preceding autophagosome synthesis which is important for the recruitment of other Atg proteins [21,29,30,31]. Our study confirmed that *BECN1* gene was activated post-effort, suggesting an important role of this protein in inducing an autophagic response following high intensity exercise. It is worth noting that anaerobic effort caused significantly higher increases in the expression of the analyzed genes than aerobic effort.

Increased expression of the *MAP1LC3B* gene encoding LC3B protein indirectly implies the activation of the autophagosome complex [21,73]. Our study showed that both types of intense-stimulating effort induced a significant increase in the expression of *MAP1LC3B*. However, the anaerobic effort caused a significantly higher biological response based on gene expression level. The roles of Atg12-Atg5-Atg16L1 and Atg7 in the autophagosome formation complex are widely described [21,30]. It is also known that the adaptive protein, p62/SQSTM1 interacts with LC3, and is specifically degraded by the autophagous-lysosomal pathway [31]. This phenomenon causes the accumulation of p62 in the cell and hence the activation of the NF-κB signaling pathway through its binding site with the TRAF6 receptor [74]. This signaling pathway is known to activate autophagy; however, dysfunction in the autophagy process may alter the signal transduction pathway and induce apoptosis [75].

The post-effort and short-term recovery (lactate recovery) gene expression levels we observed indicate probable involvement of this mechanism in autophagy induction as a result of endurance and speed efforts. In the case of the *ATG* genes (*ATG5*, *ATG7*, *ATG12*, *ATG16L1*), relative expression levels were significantly higher after the anaerobic physical effort. Upregulation of p62 is known to positively control apoptosis through polyubiquitination and aggregation of the key initiator caspase-8 [76], thus playing a potential role in cross-regulation between autophagy and apoptosis.

Weng et al. [77] demonstrated that hypoxic exercise suppressed autophagy in CD4+ lymphocytes during the initiation, elongation, maturation and fusion phases, observing a decrease in the expression of *BECN1*, *Atg-1*, *MAP1LC3B*, *Atg-12* and *LAMP2* genes. Simultaneously, apoptosis increased as a result of increased concentrations of phospho-Bcl-2 and active caspase 3 and 9 in lymphocytes [77]. However, an adaptive response to physical exercise was shown to affect the activation of autophagy, as both interval and continuous physical training for 5 weeks effectively increased the expression of Beclin-1, and thus weakened the reduction in autophagy caused by hypoxia [77]. These results, however, are restricted to CD4+ lymphocytes, and in our study we assessed change in gene expression across the entire leukocyte pool. It should be pointed out that intense exercise favorably mobilizes NK cells and cytotoxic (CD8+) T-lymphocytes, which show a high level of cytotoxicity and potential for migration into tissues. Moreover, neutrophils and monocytes are the cells responsible for infiltration and regeneration of muscles after exercise [68,78,79].

Activation of the autophagy process during exercise has been well documented in skeletal muscles [32,80,81,82,83,84]. Proper induction of autophagy during muscle contraction has been shown to be important in maintaining cellular energy homeostasis [32,80]. Autophagy biomarkers tested in skeletal muscle biopsies are upregulated after ultra-endurance running [83] or high-intensity cycling [84]. It has been shown that *BECN1*, *BNIP3* and *ATG12* expression increases in human skeletal muscle in response to acute high-endurance exercise [83]. However, a study of LC3 in response to acute endurance running showed a significant increase [83], and in other studies with shorter exercise durations, a decrease in LC3II protein content in human skeletal muscle was observed [81,84]. Moreover, Masschelein et al. [85] did not find any change in LC3II protein content after acute endurance exercise although there were differences in p62 protein content after different types of exercise. After acute endurance exercise (of high intensity), a decrease in p62 content was shown [84], while no changes in this protein level were observed after low-intensity endurance exercise [81,82,83]. The abovementioned results of studies investigating the influence of exercise on autophagy-related gene expression and protein levels suggested that the autophagic response is intensity-dependent. It seems to be consistent with the observations of our study at least at the gene expression level.

We have evidenced that the duration of the physical activity and its bioenergetic cost (aerobic or anaerobic) has an important impact on the degree of increase in gene expression of selected autophagy-related genes. Taking into account the protective role of the autophagy process, as demonstrated during acute exercise in the heart muscle, in which mitophagy is activated by reducing inflammation caused by mitochondrial stress, to minimize myocardial damage and improve the function of the cardiovascular system [32,86], may explain the stronger impact of anaerobic effort on the expression of these genes.

## 5. Conclusions

The intense efforts induced by the progressive endurance test protocol and anaerobic speed test caused an increase in the expression of genes encoding chemokines involved in the invasion and migration of immune cells. The increased expression of genes encoding pro- and anti-inflammatory cytokines suggest that both aerobic (endurance) and anaerobic (speed) efforts shift the immune balance via the cell death mechanism, at least at the gene expression level. The relative expression of genes encoding IL-2 and IL-4 indicate a shift in the molecular mechanism to pathways involving IL-4 and INF-γ. This probably explains the significant increase in the expression of pro-apoptotic genes such as *BAX*, and anti-apoptotic genes, e.g., *BCL2*.

It seems that the duration of physical activity and its bioenergetic cost has an important impact on the level of the expression of studied autophagy-related genes. Knowing that anaerobic effort is more strenuous and requires a higher bioenergetic investment, this may explain the stronger impact of anaerobic effort on the expression of the observed genes.

The main limitation of the study is analyzing only the changes in gene expression level changes that have little insight into autophagy process as described by Klionsky et al. [87]. More extended study including not only gene expression but protein level or preferably proteome and metabolome analysis is required to examine the influence of aerobic and anaerobic exercise on autophagy process in young men. Another important limitation of the study is that it was performed on a limited number of participants and only males. The reason for excluding women from the study was to avoid the possible influence of hormone changes during women’s menstrual cycle. However, adding a group of women would significantly enrich the future study.

## Figures and Tables

**Table 1:** A baseline characteristic of the participants.

Parameter	(*n* = 35)
Age (years)	19 (16–21)
Height (cm)	181 ± 7
Weight (kg)	75.3 ± 9.1
BMR (kJ)	8480 ± 870
FAT (%)	10.8 ± 3.5
FAT MASS (kg)	8.36 ± 3.28
FFM (kg)	66.98 ± 6.78
TWB (kg)	49.0 ± 4.9
VO_2_max (mL/kg/min)	61.7 ± 5.4
HR_max_ (beats/min)	198 ± 8
V_E_ (L/min)	144.1 ± 23.7
AT (beats/min)	166 ± 13
MVV (L/min)	186 ± 17
MET (mL/kg/min)	17.8 ± 1.5
Rf	60.8 ± 7.6

The table presents mean ± standard deviation values (except for the age, where median (min–max) is presented) characterizing the participants. Body composition parameters were determined using Body Composition Analyzer Tanita BC-418MA (Tanita, Tokyo, Japan), while cardiorespiratory parameters were determined using the state-of-the-art breath by breath gas exchange data analyzer Quark CPET (Cosmed, Albano Laziale, Italy). *n*—number of participants, BMR—basal metabolic rate, FAT—percentage of fat, FFM—fat free mass, TBW—total body water, VO_2_max—maximum oxygen uptake; HRmax—maximum heart rate; AT—anaerobic threshold; VE—minute ventilation; MVV—maximal voluntary ventilation; MET—metabolic equivalent; Rf—respiratory frequency.

**Table 2:** Primers used for qPCR in the study.

Gene	Forward Primer	Reverse Primer	Amplicon Lenght (bp)	TM of the Amplification Products (°C)
*CXCL5*	AGACCACGCAAGGAGTTCATC	GTTTTCCTTGTTTCCACCGTCC	185	81.5
*CXCL8*	AGGAAGAAACCACCGGAAGG	GGCAAAACTGCACCTTCACA	119	82.5
*CXCL9*	TGGTGTTCTTTTCCTCTTGGG	TCTCACTACTGGGGTTCCTTG	70	78
*CXCL10*	AGTGGCATTCAAGGAGTACCT	CGTGGACAAAATTGGCTTGC	128	77.5
*CXCL11*	ATAGGCCCTGGGGTAAAAGC	CTTGCTTGCTTCGATTTGGGA	152	77.5
*CXCL12*	CCGCACTTTCACTCTCCGTC	CAGCACGACCACGACCTT	118	77.5
*IL2*	CAGCTACAACTGGAGCATTTACT	TTCAGTTCTGTGGCCTTCTTG	131	86
*IL4*	CCATGAGAAGGACACTCGCT	CGTACTCTGGTTGGCTTCCTT	151	86
*IL6*	GTGAAAGCAGCAAAGAGGCAC	GATTTTCACCAGGCAAGTCTCC	113	79.5
*IL10*	CTTCCCTGTGAAAACAAGAGCA	ACTCATGGCTTTGTAGATGCCT	90	78
*IL17A*	TCTCATAGCAGGCACAAACTCA	GCAGTAGCAGTGACACCAATG	92	79
*IFNG*	TGAAGAATTGGAAAGAGGAGAGTG	TCTCCACACTCTTTTGGATGC	117	74.5
*TNF*	AGCCCATGTTGTAGCAAACCC	GGACCTGGGAGTAGATGAGGT	149	87
*BCL2*	CGCGACTCCTGATTCATTGG	CAGTCTACTTCCTCTGTGATGTTGT	165	77.5
*BAX*	GCCCTTTTCTACTTTGCCAGC	CGGAGGAAGTCCAATGTCCA	101	83
*BNIP3*	CACGAGCGTCATGAAGAAAGG	GACGCCTTCCAATATAGATCCCCAA	119	79.5
*BECN1*	CCAGATGCGTTATGCCCAGA	TCCATTCCACGGGAACACTG	146	83
*MAP1LC3B*	GACCGCTGTAAGGAGGTACA	CAGCTGCTTCTCACCCTTGT	90	83.5
*ATG5*	TTGGGCCATCAATCGGAAAC	AGTGTGTGCAACTGTCCATCT	150	78.5
*ATG7*	CTGAACGAGTATCGGCTGGA	AGTGTTCCAATAGCTGGGCA	158	83.5
*ATG12*	CCCCAGACCAAGAAGTTGGA	TTCAGAGCTGTCTCTTCCGTG	155	79
*ATG16L1*	GATTACGGCACACACTCACG	TGCTGCGTAGATCCCAGAGT	123	83.5
*SQSTM1*	TGTCCCTGAAAGAGAAGATGGC	CCCTCAAAATCAAAGCCTGTCC	155	87
*RACK1*	GAGTGTGGCCTTCTCCTCTG	GCTTGCAGTTAGCCAGGTTC	224	84.5

**Table 3:** The results of the tests performed by the participants and the level of lactic acid.

	Beep Test	RSA Test	Mann–Whitney *p*-Value
Test Results (Beep decimal score or RSA mean score (s), respectively)	13.2 (11.3–14.6)	2.73 (2.15–3.27)	
LA (mmol/L)			
Friedman’s ANOVA *p*-value	<0.0001	<0.0001	
pre-test	2.2 (1.9–2.4) ^aaaa^	3.1 (2.9–3.4) ^aaaa^	<0.0001
post-test	8.1 (7.5–8.6) ^bbbb^	15.2 (12.6–16.4) ^bbbb^	<0.0001
LA-rec	2.1 (1.8–2.3)	2.9 (2.8–3.2) ^c^	<0.0001
post-test/pre-test ratio	3.73 (3.23–4.26)	4.74 (4.13–5.25)	<0.0001
LA-rec/pre-test ratio	0.95 (0.91–1.00)	0.93 (0.91–0.97)	0.3743

The table presents median (interquartile range). Significance levels of differences observed between analyzed time points (pre-test vs. post-test vs. LA-rec) were assessed using Friedman’s analysis of variance for repeated measures followed by post hoc Dunn’s test with Bonferroni correction. Post hoc *p*-values: ^aaaa^ *p* < 0.0001 for pre-test vs. post-test, ^bbbb^ *p* < 0.0001 for post-test vs. LA-rec, ^c^ *p* < 0.05 for pre-test vs. LA-rec. Significance levels of differences observed between variables found for Beep vs. RSA tests were assessed using the U Mann–Whitney test. Beep—maximal multistage 20-m shuttle run test, LA—lactic acid, RSA—repeated speed ability test. The analyses were performed before (baseline, pre-test) and after the effort (5 min post-effort, post-test and during lactate recovery time about 1 h after the test, LA-rec).

**Table 4:** The expression (2^−ΔCt^ values) of genes encoding selected chemokines of studied participants’ blood cells.

Gene		Beep Test	RSA Test	Mann–Whitney *p*-Value
*CXCL5*	Friedman’s ANOVA *p*-value	<0.0001	<0.0001	
	pre-test	0.017 ^aaaa^ (0.011–0.028)	0.007 ^aaaa^ (0.005–0.010)	<0.0001
	post-test	0.058 ^bbbb^ (0.030–0.071)	0.027 ^bb^ (0.019–0.038)	0.0001
	LA-rec	0.016 (0.012–0.026)	0.037 ^cccc^ (0.026–0.051)	<0.0001
	post-test/pre-test ratio	2.68 (2.20–3.65)	3.58 (2.66–4.88)	0.00569
	LA-rec/pre-test ratio	0.92 (0.76–1.24)	4.86 (3.41–6.22)	<0.0001
*CXCL8*	Friedman’s ANOVA *p*-value	<0.0001	<0.0001	
	pre-test	0.005 ^aaaa^ (0.003–0.006)	0.003 ^aaaa^ (0.002–0.006)	<0.0001
	post-test	0.009 ^bb^ (0.007–0.014)	0.014 ^bbbb^ (0.008–0.027)	0.0001
	LA-rec	0.008 (0.005–0.010)	0.016 (0.009–0.028)	0.0001
	post-test/pre-test ratio	1.80 (1.40–3.05)	4.31 (3.16–5.35)	<0.0001
	LA-rec/pre-test ratio	1.51 (1.15–2.37)	4.23 (3.75–5.83)	<0.0001
*CXCL9*	Friedman’s ANOVA *p*-value	<0.0001	<0.0001	
	pre-test	0.005 ^aaaa^ (0.009–0.005)	0.004 ^aaaa^ (0.002–0.006)	0.0187
	post-test	0.015 ^bbbb^ (0.008–0.018)	0.019 ^bb^ (0.009–0.037)	0.0800
	LA-rec	0.006 (0.005–0.011)	0.010 ^cccc^ (0.006–0.016)	0.0207
	post-test/pre-test ratio	1.96 (1.78–2.99)	4.60 (4.07–5.96)	<0.0001
	LA-rec/pre-test ratio	1.12 (0.87–1.38)	2.71 (2.37–3.76)	<0.0001
*CXCL10*	Friedman’s ANOVA *p*-value	< 0.0001	< 0.0001	
	pre-test	0.004 ^aaaa^ (0.003–0.006)	0.003 ^aaaa^ (0.002–0.006)	0.4000
	post-test	0.008 ^b^ (0.006–0.009)	0.009 (0.007–0.016)	0.0632
	LA-rec	0.006 ^cccc^ (0.004–0.008)	0.009 ^cccc^ (0.007–0.016)	<0.0001
	post-test/pre-test ratio	1.73 (1.44–2.38)	2.54 (2.16–2.99)	<0.0001
	LA-rec/pre-test ratio	1.36 (1.19–1.65)	2.53 (1.91–3.55)	<0.0001
*CXCL11*	Friedman’s ANOVA *p*-value	<0.0001	<0.0001	
	pre-test	0.004 ^aaaa^ (0.003–0.004)	0.003 ^aaaa^ (0.002–0.007)	0.5206
	post-test	0.015 (0.013–0.017)	0.016 (0.010–0.025)	0.7001
	LA-rec	0.014 ^cccc^ (0.011–0.016)	0.016 ^cccc^ (0.009–0.021)	0.1976
	post-test/pre-test ratio	4.12 (3.21–5.06)	4.04 (3.23–5.41)	0.5912
	LA-rec/pre-test ratio	3.82 (2.77–4.19)	4.02 (2.83–5.73)	0.1561
*CXCL12*	Friedman’s ANOVA *p*-value	0.0419	0.2263	
	pre-test	0.004 (0.003–0.004)	0.007 (0.006–0.009)	<0.0001
	post-test	0.004 (0.003–0.004)	0.007 (0.007–0.009)	<0.0001
	LA-rec	0.003 ^c^ (0.003–0.004)	0.008 (0.007–0.010)	<0.0001
	post-test/pre-test ratio	1.12 (0.87–1.35)	1.04 (0.74–1.49)	0.9907
	LA-rec/pre-test ratio	0.94 (0.79–1.23)	1.24 (0.82–1.42)	0.0454

The table presents median (interquartile range) values. Significance levels of differences observed between analyzed time points (pre-test vs. post-test vs. LA-rec) were assessed using Friedman’s analysis of variance for repeated measures followed by post hoc Dunn’s test with Bonferroni correction. Post hoc *p*-values: ^aaaa^ *p* < 0.0001 for pre-test vs. post-test, ^b^ *p* < 0.05, ^bb^ *p* < 0.01, ^bbbb^ *p* < 0.0001 for post-test vs. LA-rec, ^c^ *p* < 0.05, ^cccc^ *p* < 0.0001 for pre-test vs. LA-rec. Significance levels of differences observed between variables found for Beep vs. RSA tests were assessed using U Mann–Whitney test. Beep—maximal multistage 20-m shuttle run test, LA—lactic acid, RSA—repeated speed ability test. The analyses were performed before (baseline, pre-test) and after the effort (5 min post-effort, post-test and during lactate recovery time about 1 h after the test, LA-rec).

**Table 5:** The expression (2^−ΔCt^ values) of genes encoding selected cytokines of studied participants’ blood cells.

Gene		Beep Test	RSA Test	Mann–Whitney *p*-Value
*IL2*	Friedman’s ANOVA *p*-value	<0.0001	<0.0001	
	pre-test	0.004 ^aaaa^ (0.003–0.004)	0.004 ^aaaa^ (0.003–0.005)	0.8610
	post-test	0.008 ^bbbb^ (0.007–0.009)	0.025 ^bbb^ (0.021–0.035)	<0.0001
	LA-rec	0.003 (0.003–0.004)	0.016 ^cccc^ (0.009–0.020)	<0.0001
	post-test/pre-test ratio	2.04 (1.84–2.47)	7.38 (4.82–9.43)	<0.0001
	LA-rec/pre-test ratio	0.90 (0.71–1.12)	3.93 (2.70–4.92)	<0.0001
*IL4*	Friedman’s ANOVA *p*-value	<0.0001	<0.0001	
	pre-test	0.004 ^aaaa^ (0.003–0.004)	0.003 ^aaaa^ (0.002–0.004)	0.1187
	post-test	0.014 ^bb^ (0.011–0.016)	0.014 (0.009–0.017)	0.9071
	LA-rec	0.008 ^cccc^ (0.008–0.010)	0.013 ^cccc^ (0.010–0.017)	<0.0001
	post-test/pre-test ratio	3.54 (2.81–4.90)	4.40 (3.21–6.05)	0.1303
	LA-rec/pre-test ratio	2.20 (1.88–2.71)	4.12 (3.19–5.39)	<0.0001
*IL6*	Friedman’s ANOVA *p*-value	<0.0001	<0.0001	
	pre-test	0.002 ^aaaa^ (0.002–0.002)	0.003 ^aaaa^ (0.001–0.005)	0.1215
	post-test	0.015 ^bbb^ (0.012–0.018)	0.019 ^b^ (0.011–0.030)	0.1244
	LA-rec	0.009 ^cccc^ (0.008–0.010)	0.014 ^cccc^ (0.008–0.031)	0.0036
	post-test/pre-test ratio	6.75 (5.88–8.60)	8.05 (5.04–11.88)	0.2060
	LA-rec/pre-test ratio	4.14 (3.52–4.97)	6.56 (4.47–9.33)	<0.0001
*IL10*	Friedman’s ANOVA *p*-value	<0.0001	<0.0001	
	pre-test	0.004 ^aaaa^ (0.003–0.005)	0.011 ^aaaa^ (0.006–0.027)	<0.0001
	post-test	0.008 (0.007–0.009)	0.068 ^b^ (0.031–0.107)	<0.0001
	LA-rec	0.008 ^cccc^ (0.006–0.009)	0.042 ^cccc^ (0.019–0.092)	<0.0001
	post-test/pre-test ratio	2.05 (1.76–2.59)	5.15 (3.27–7.73)	<0.0001
	LA-rec/pre-test ratio	1.82 (1.47–2.85)	4.24 (2.68–5.45)	<0.0001
*IL17A*	Friedman’s ANOVA *p*-value	0.20103	0.24660	
	pre-test	0.029 (0.026–0.036)	0.001 (0.001–0.002)	<0.0001
	post-test	0.028 (0.023–0.032)	0.001 (0.001–0.002)	<0.0001
	LA-rec	0.031 (0.025–0.036)	0.001 (0.001–0.001)	<0.0001
	post-test/pre-test ratio	0.94 (0.74–1.04)	1.14 (0.84–1.38)	0.0274
	LA-rec/pre-test ratio	0.98 (0.76–1.42)	1.12 (0.89–1.41)	0.3033
*IFNG*	Friedman’s ANOVA *p*-value	<0.0001	<0.0001	
	pre-test	0.031 ^aaaa^ (0.025–0.034)	0.027 ^aaaa^ (0.020–0.037)	0.4268
	post-test	0.100 (0.091–0.121)	0.128 (0.103–0.149)	0.0522
	LA-rec	0.118 ^cccc^ (0.096–0.138)	0.104 ^cccc^ (0.075–0.151)	0.3870
	post-test/pre-test ratio	3.60 (2.94–4.60)	4.84 (3.55–5.89)	0.0061
	LA-rec/pre-test ratio	3.89 (2.92–5.00)	3.58 (2.43–5.72)	0.5282
*TNF*	Friedman’s ANOVA *p*-value	<0.0001	<0.0001	
	pre-test	0.003 ^aaaa^ (0.003–0.004)	0.004 ^aaaa^ (0.002–0.006)	0.5359
	post-test	0.008 (0.006–0.009)	0.015 (0.008–0.032)	<0.0001
	LA-rec	0.007 ^cccc^ (0.006–0.009)	0.012 ^cccc^ (0.007–0.021)	0.0003
	post-test/pre-test ratio	2.19 (1.81–2.53)	4.07 (2.97–5.00)	<0.0001
	LA-rec/pre-test ratio	1.93 (1.60–2.80)	3.00 (2.25–0.40)	<0.0001

The table presents median (interquartile range) values. Significance levels of differences observed between analyzed time points (pre-test vs. post-test vs. LA-rec) were assessed using Friedman’s analysis of variance for repeated measures followed by post hoc Dunn’s test with Bonferroni correction. Post hoc *p*-values: ^aaaa^ *p* < 0.0001 for pre-test vs. post-test, ^b^ *p* < 0.05, ^bb^ *p* < 0.01, ^bbb^ *p* < 0.001, ^bbbb^ *p* < 0.0001 for post-test vs. LA-rec, ^cccc^ *p* < 0.0001 for pre-test vs. LA-rec. Significance levels of differences observed between variables found for Beep vs. RSA tests were assessed using U Mann–Whitney test. Beep—maximal multistage 20-m shuttle run test, IFNG—interferon gamma, IL—interleukin, LA—lactic acid, RSA—repeated speed ability test, TNF—tumor necrosis factor alpha. The analyses were performed before (baseline, pre-test) and after the effort (5 min post-effort, post-test and during lactate recovery time about 1 h after the test, LA-rec).

**Table 6:** The expression (2^−ΔCt^ values) of death-related genes of studied participants’ blood cells.

Gene		Beep Test	RSA Test	Mann–Whitney *p*-Value
*BCL2*	Friedman’s ANOVA *p*-value	<0.0001	<0.0001	
	pre-test	0.016 ^aaaa^ (0.011–0.024)	0.010 ^aaaa^ (0.007–0.015)	0.0002
	post-test	0.036 ^bb^ (0.025–0.062)	0.050 (0.029–0.059)	0.3033
	recovery	0.029 ^cc^ (0.018–0.033)	0.050 ^cccc^ (0.032–0.079)	0.0001
	post-test/pre-test ratio	3.73 (3.23–4.26)	4.74 (4.13–5.25)	<0.0001
	LA-rec/pre-test ratio	0.95 (0.91–1.00)	4.53 (3.44–6.98)	<0.0001
*BAX*	Friedman’s ANOVA *p*-value	<0.0001	<0.0001	
	pre-test	0.061 ^aaaa^ (0.041–0.070)	0.038 ^aaaa^ (0.029–0.075)	0.0685
	post-test	0.233 ^bb^ (0.120–0.306)	0.464 ^bb^ (0.318–0.559)	<0.0001
	recovery	0.119 ^cccc^ (0.097–0.154)	0.186 ^cccc^ (0.103–0.328)	0.0266
	post-test/pre-test ratio	3.77 (2.63–5.09)	11.33 (6.45–15.08)	<0.0001
	LA-rec/pre-test ratio	2.05 (1.63–3.00)	3.89 (2.92–6.14)	<0.0001
*BNIP3*	Friedman’s ANOVA *p*-value	<0.0001	<0.0001	
	pre-test	0.014 ^aaaa^ (0.009–0.018)	0.009 ^aaaa^ (0.004–0.013)	0.0006
	post-test	0.046 ^bbbb^ (0.030–0.058)	0.066 ^bbb^ (0.049–0.089)	0.0005
	recovery	0.011 (0.007–0.017)	0.031 ^cccc^ (0.020–0.052)	<0.0001
	post-test/pre-test ratio	3.46 (2.57–3.87)	8.05 (6.58–11.57)	<0.0001
	LA-rec/pre-test ratio	0.95 (0.45–1.50)	4.09 (3.32–6.00)	<0.0001
*BECN1*	Friedman’s ANOVA *p*-value	<0.0001	<0.0001	
	pre-test	0.027 ^aaaa^ (0.022–0.039)	0.026 ^aaaa^ (0.019–0.039)	0.6321
	post-test	0.073 (0.055–0.095)	0.078 (0.059–0.121)	0.6405
	recovery	0.062 ^cccc^ (0.053–0.084)	0.082 ^cccc^ (0.068–0.113)	0.0176
	post-test/pre-test ratio	2.61 (2.05–3.09)	2.97 (2.48–3.84)	0.0250
	LA-rec/pre-test ratio	2.32 (1.89–2.86)	3.12 (2.33–4.42)	0.0015
*MAP1LC3B*	Friedman’s ANOVA *p*-value	<0.0001	<0.0001	
	pre-test	0.003 ^aaaa^ (0.002–0.005)	0.003 ^aaaa^ (0.002–0.005)	0.6156
	post-test	0.010 (0.005–0.014)	0.015 ^b^ (0.009–0.022)	0.0085
	recovery	0.008 ^cccc^ (0.005–0.014)	0.012 ^cccc^ (0.006–0.016)	0.3318
	post-test/pre-test ratio	2.71 (1.97–3.60)	5.27 (3.92–6.49)	<0.0001
	LA-rec/pre-test ratio	2.91 (1.93–3.77)	3.32 (2.47–5.27)	0.0381
*ATG5*	Friedman’s ANOVA *p*-value	<0.0001	<0.0001	
	pre-test	0.040 ^aaaa^ (0.027–0.058)	0.027 ^aaaa^ (0.019–0.039)	0.0220
	post-test	0.074 (0.050–0.128)	0.115 (0.083–0.182)	0.0019
	recovery	0.070 ^cccc^ (0.054–0.090)	0.108 ^cccc^ (0.074–0.152)	0.0042
	post-test/pre-test ratio	2.21 (1.61–2.71)	4.71 (3.52–5.81)	<0.0001
	LA-rec/pre-test ratio	1.91 (1.44–.43)	3.88 (2.72–5.45)	<0.0001
*ATG7*	Friedman’s ANOVA *p*-value	<0.0001	<0.0001	
	pre-test	0.027 ^aaaa^ (0.019–0.039)	0.021 ^aaaa^ (0.011–0.037)	0.1003
	post-test	0.071 ^bbbb^ (0.036–0.103)	0.138 ^b^ (0.076–0.237)	<0.0001
	recovery	0.031 (0.019–0.038)	0.094 ^cccc^ (0.059–0.140)	<0.0001
	post-test/pre-test ratio	2.32 (1.65–3.00)	6.38 (4.47–10.89)	<0.0001
	LA-rec/pre-test ratio	0.95 (0.76–1.26)	4.15 (3.19–5.79)	<0.0001
*ATG12*	Friedman’s ANOVA *p*-value	<0.0001	<0.0001	
	pre-test	0.019 ^aaaa^ (0.012–0.035)	0.006 ^aaaa^ (0.004–0.012)	<0.0001
	post-test	0.060 (0.044–0.138)	0.038 ^bb^ (0.018–0.065)	0.0007
	recovery	0.074 ^cccc^ (0.040–0.105)	0.022 ^cccc^ (0.009–0.040)	<0.0001
	post-test/pre-test ratio	3.91 (3.09–4.86)	5.70 (3.66–8.46)	0.0003
	LA-rec/pre-test ratio	3.25 (2.38–4.58)	3.94 (2.33–4.83)	0.7615
*ATG16L1*	Friedman’s ANOVA *p*-value	<0.0001	<0.0001	
	pre-test	0.023 ^aaaa^ (0.015–0.030)	0.009 ^aaaa^ (0.004–0.015)	<0.0001
	post-test	0.099 ^bbb^ (0.051–0.129)	0.075 ^b^ (0.036–0.136)	0.3202
	recovery	0.040 ^cccc^ (0.030–0.061)	0.051 ^cccc^ (0.021–0.069)	0.5831
	post-test/pre-test ratio	4.19 (3.35–5.61)	7.76 (5.53–11.09)	<0.0001
	LA-rec/pre-test ratio	1.97 (1.41–2.54)	5.23 (3.28–7.49)	<0.0001
*SQSTM1*	Friedman’s ANOVA *p*-value	<0.0001	<0.0001	
	pre-test	0.016 ^aaaa^ (0.009–0.023)	0.019 ^aaaa^ (0.011–0.041)	0.0800
	post-test	0.033 (0.021–0.062)	0.168 (0.085–0.257)	<0.0001
	recovery	0.032 ^cccc^ (0.022–0.045)	0.140 ^cccc^ (0.078–0.204)	<0.0001
	post-test/pre-test ratio	2.24 (2.00–2.90)	6.30 (5.02–9.13)	<0.0001
	LA-rec/pre-test ratio	1.91 (1.63–.80)	5.48 (4.26–9.27)	<0.0001

The table presents median (interquartile range) values. Significance levels of differences observed between analyzed time points (pre-test vs. post-test vs. LA-rec) were assessed using Friedman’s analysis of variance for repeated measures followed by post hoc Dunn’s test with Bonferroni correction. Post hoc *p*-values: ^aaaa^ *p* < 0.0001 for pre-test vs. post-test, ^b^ *p* < 0.05, ^bb^ *p* < 0.01, ^bbb^ *p* < 0.001, ^bbbb^ *p* < 0.0001 for post-test vs. LA-rec, ^cc^ *p*< 0.01 ^cccc^ *p* < 0.0001 for pre-test vs. LA-rec. Significance levels of differences observed between variables found for Beep vs. RSA tests were assessed using U Mann–Whitney test. Beep—maximal multistage 20-m shuttle run test, LA—lactic acid, RSA—repeated speed ability test. The analyses were performed before (baseline, pre-test) and after the effort (5 min post-effort, post-test and during lactate recovery time about 1 h after the test, LA-rec).

## Data Availability

The raw data of this study can be made available by the corresponding author upon request.

## Supplementary Materials

The following are available online at https://www.mdpi.com/article/10.3390/cells10061406/s1. Figure S1: Gel representative of the size of the amplification products. 2% agarose gel in 1× TAE buffer was run for 30 min at 120V. Gel Red dye was used to visualize amplification products and molecular weight size marker.
