# Supplementary material for: Post-Effort Changes in Autophagy- and Inflammation-Related Gene Expression in White Blood Cells of Healthy Young Men
Source: Cells. 2021 Jun 6;10(6):1406. doi: 10.3390/cells10061406 (PMC8229752; doi:10.3390/cells10061406)
Supplement: Supplementary file 1 [file cells-10-01406-s001.zip › cells-1215024-supplementary.pdf]

# Supplementary Materials

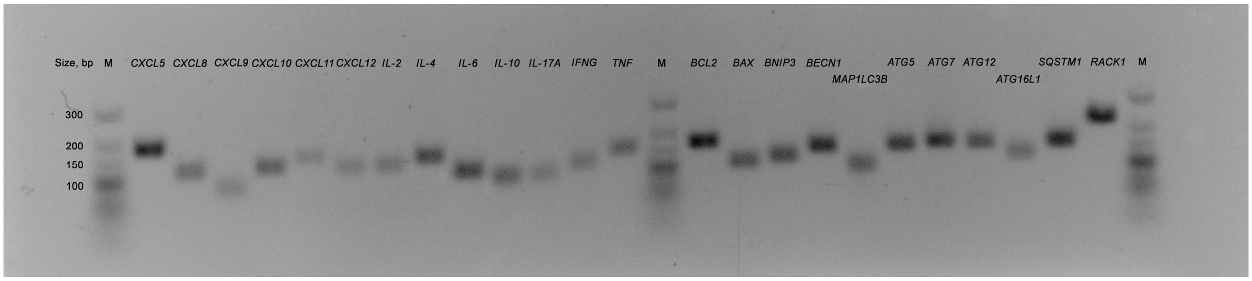


**Figure S1.** Gel representative of the size of the amplification products. 2% agarose gel in 1x TAE buffer was run for 30 min. at 120V. Gel Red dye was used to visualize amplification products and molecular wight size marker.
